# An epoxide hydrolase from endophytic *Streptomyces* shows unique structural features and wide biocatalytic activity

**DOI:** 10.1107/S2059798320010402

**Published:** 2020-08-17

**Authors:** Gabriela D. Tormet-González, Carolina Wilson, Gabriel Stephani de Oliveira, Jademilson Celestino dos Santos, Luciana G. de Oliveira, Marcio Vinicius Bertacine Dias

**Affiliations:** a Department of Organic Chemistry, Institute of Chemistry, University of Campinas, Campinas-SP 3083-970, Brazil; b Department of Microbiology, Institute of Biomedical Science, University of São Paulo, Avenida Prof. Lineu Prestes 1374, São Paulo-SP 05508-000, Brazil; c Department of Biology, IBILCE – University of State of São Paulo, São José do Rio Preto-SP 15054-000, Brazil; d Department of Chemistry, University of Warwick, Warwick CV4 7AL, United Kingdom

**Keywords:** epoxide hydrolases, α/β-hydrolases, biocatalysis, *Streptomyces*

## Abstract

The genus *Streptomyces* is characterized by the production of a wide variety of secondary metabolites with remarkable biological activities and broad antibiotic capabilities. The presence of an unprecedented number of genes encoding hydrolytic enzymes with industrial appeal such as epoxide hydrolases (EHs) reveals its resourceful microscopic machinery. The whole-genome sequence of *Streptomyces* sp. CBMAI 2042, an endophytic actinobacterium isolated from *Citrus sinensis* branches, was explored by genome mining, and a putative α/β-epoxide hydrolase named B1EPH2 and encoded by 344 amino acids was selected for functional and structural studies. The crystal structure of B1EPH2 was obtained at a resolution of 2.2 Å and it was found to have a similar fold to other EHs, despite its hexameric quaternary structure, which contrasts with previously solved dimeric and monomeric EH structures. While B1EPH2 has a high sequence similarity to EHB from *Mycobacterium tuberculosis*, its cavity is similar to that of human EH. A group of 12 aromatic and aliphatic racemic epoxides were assayed to determine the activity of B1EPH2; remarkably, this enzyme was able to hydrolyse all the epoxides to the respective 1,2-diols, indicating a wide-range substrate scope acceptance. Moreover, the (*R*)- and (*S*)-enantiomers of styrene oxide, epichlorohydrin and 1,2-epoxybutane were used to monitor enantiopreference. Taken together, the functional and structural analyses indicate that this enzyme is an attractive biocatalyst for future biotechnological applications.

## Abstract 2

The crystal structure of an epoxide hydrolase from *Streptomyces* sp. CBMAI 2042 was determined and revealed the conserved overall fold found in other α/β-hydrolases, despite its unusual hexameric quaternary structure. Although its primary sequence is similar to that of a *Mycobacterium tuberculosis* epoxide hydrolase, its active-site architecture, and particularly its volume, closely resembles the human epoxide hydrolase.

## 1. Introduction   

Epoxides and the associated enantiopure epoxides and diols are high-value compounds (de Vries & Janssen, 2003 ▸; Archelas & Furstoss, 2001 ▸; Kotik *et al.*, 2010 ▸) that are in demand as fine chemicals by the pharmaceutical, biotechnological and chemical industries owing to their applicability as precursors and intermediates in the synthesis of a large number of organic compounds (Saini & Sareen, 2017 ▸; Fretland & Omiecinski, 2000 ▸; Savle *et al.*, 1998 ▸; Archelas & Furstoss, 2001 ▸). Electrophilic functional groups such as epoxides are widespread in nature and are essential for the biological activity of natural products targeting nucleophilic centers in macromolecules in the living world.

Soluble epoxide hydrolases (EHs) are a class of enzymes that can promote the asymmetric hydrolysis of epoxides to their respective 1,2-diols and have emerged as versatile biocatalysts for the production of chiral intermediates (Saini & Sareen, 2017 ▸, 2019 ▸; Pedragosa-Moreau *et al.*, 1995 ▸; Faber, 2011 ▸; Archelas & Furstoss, 2001 ▸). Microbial EHs have been widely used as biocatalysts in diverse organic synthesis routes owing to their stability, broad substrate acceptance and advantages of chemoselectivity, regioselectivity and enantio­selectivity. Furthermore, EHs represent a green alternative to the classical asymmetric synthesis of epoxides and diols (Hammock *et al.*, 1997 ▸; Morisseau & Hammock, 2005 ▸; Horsman *et al.*, 2013 ▸). For instance, EHs are suitable for chemoenzymatic approaches to prepare (*S*)-β-amino alcohols through an epoxide hydrolase/alcohol dehydrogenase/transaminase cascade (Zhang *et al.*, 2019 ▸) or to prepare (2*R*,5*R*)-linalool oxide using various EHs and a Tsuji–Trost cyclo­etherification (van Lint *et al.*, 2019 ▸).

EHs are ubiquitous in nature and play various roles in the physiologies of all organisms, including the detoxification of xenobiotics (Decker *et al.*, 2009 ▸), regulation of blood pressure (Sinal *et al.*, 2000 ▸) and inflammatory responses (Morisseau & Hammock, 2005 ▸). Epoxide hydrolases are classified into two different groups based on their folds: soluble α/β-hydrolases (EC 3.3.2.10), which are the most diverse and the most studied and are already used for transformations in industry (Pedragosa-Moreau *et al.*, 1996 ▸), and limonene epoxide hydrolases (LEHs; EC 3.3.2.8), which differ from other EHs in their fold and in their one-step catalytic mechanism (Arand *et al.*, 2003 ▸). Enzymes similar to LEHs are known to be involved in the biosynthesis of several ionophore polyethers (Dutton *et al.*, 1995 ▸).

Structurally, the EHs that belong to the α/β-hydrolases consist of two dissimilar N- and C-terminal domains. The C-terminal domain (hydrolase domain) is well conserved and is composed of an eight-stranded β-sheet flanked by α-helices and by the N-terminal domain (cap domain), which is less conserved and is generally formed by a bundle of α-helices (Nardini *et al.*, 2001 ▸; Argiriadi *et al.*, 1999 ▸). The EH active site is usually located in a groove between the hydrolase and cap domains and has a catalytic triad formed by two acidic residues and a nucleophile, with a histidine playing the role of a general base. Two tyrosines from the cap domain are involved in the oxyanion-binding site for epoxide substrates (Nardini *et al.*, 1999 ▸, 2001 ▸; Zou *et al.*, 2000 ▸; Argiriadi *et al.*, 1999 ▸). Only a few EHs have a characterized three-dimensional structure, which supports the need for further structural studies in order to understand the basis for enantioselectivity and improved catalytic efficiency. However, most of the available EH structures are from fungi and there is a lack of structures of *Streptomyces* EHs, which differ from those involved in the biosynthesis of particular bioactive compounds (such as those similar to LEH). Importantly, the identification of microbial EHs using alternative screening strategies such as genome mining is convenient in order to overcome these limitations, and the expansion of publicly available genome databases has revealed these bacterial groups to be prolific producers of hydrolytic enzymes (Davids *et al.*, 2013 ▸; Guérard-Hélaine *et al.*, 2015 ▸; Saini & Sareen, 2019 ▸).

Our recent genome-mining studies revealed *Streptomyces* sp. CBMAI 2042, an endophytic strain isolated from *Citrus sinensis* branches, to be a promising source of secondary metabolites (de Oliveira, Sigrist *et al.*, 2019 ▸; Paulo, Sigrist, Angolini & De Oliveira, 2019 ▸; Sigrist, Paulo *et al.*, 2020 ▸; Sigrist, Luhavaya *et al.*, 2020 ▸; Paulo, Sigrist, Angolini, Eberlin *et al.*, 2019 ▸). Additionally, biotransformation experiments using whole cells revealed the capability of the strain to promote epoxide hydrolysis (unpublished results). This actinobacterium was completely sequenced using Illumina technology (GenBank RCOL00000000; de Oliveira *et al.*, 2019 ▸); two EHs were predicted from the draft genome and were named B1EPH1 and B1EPH2. Here, we describe functional and structural studies of B1EPH2, which provide novel insights into the oligomeric state of this class of proteins and also their promiscuity for alternative substrates.

## 2. Materials and methods   

### 2.1. Cloning, expression and purification of B1EPH2   

The *b1eph2* ORF was amplified from *Streptomyces* sp. CBMAI 2042 genomic DNA by PCR. B1EPH2 was annotated from whole-genome sequencing of *Streptomyces* sp. CBMAI 2042 (RCOL01000001.1:3543330..3544202; Sequence ID RLV67613.1) as a putative α/β-epoxide hydrolase encoded by 344 amino acids (Tormet-Gonzalez & de Oliveira, 2018*a* ▸,*b* ▸). The resulting strain, *Escherichia coli* DSM 32387, was deposited in the Leibniz Institute DSMZ (German Collection of Microorganisms and Cell Cultures). The encoded ORF was cloned into pET-29b(+) and the resulting plasmid pGTEPH2 was successfully transformed into *E. coli* BL21(DE3) cells, which were grown in LB medium containing 50 µg ml^−1^ kanamycin. Further data on cloning are given in Table 1 ▸. Expression of the *b1eph2* gene was induced by 0.4 m*M* IPTG for 16 h at 18°C. The cells were harvested by centrifugation and disrupted by sonication. The soluble and insoluble fractions were separated by centrifugation at 4°C. B1EPH2 was purified from the soluble fraction using an IMAC Nickel HiTrap column, pre-equilibrated with 50 m*M* HEPES pH 8.0, 300 m*M* NaCl, 10% glycerol (buffer *A*), coupled to an ÄKTA purifier system (GE) using a linear gradient of buffer *A* plus 500 m*M* imidazole (buffer *B*). A further step of gel filtration was performed using 50 m*M* HEPES pH 8.0, 300 m*M* NaCl (buffer *C*). The sample was concentrated to 15 mg ml^−1^, flash-frozen in liquid nitrogen and stored at −80°C until use.

### 2.2. Activity and enantiopreference assays   

The activity of the enzyme as epoxide hydrolase was assayed using the adrenaline test (Fluxá *et al.*, 2008 ▸) against 12 epoxide substrates dissolved in dimethyl sulfoxide (DMSO). The adrenaline test is widely used to allow the quantification of periodate-sensitive hydrolysis reactions by back-titration of the oxidant with adrenaline to produce adrenochrome as a detectable red product at λ_max_ = 490 nm. The test was prepared in 96-well microplates using enzyme concentrations between 1 and 25 µg ml^−1^ in sodium phosphate buffer pH 6.0. Negative controls relating to the same reaction without enzyme (to reflect spontaneous epoxide hydrolysis under the reaction conditions) or substrate and a positive control (1,2-cyclohexanodiol as substrate) were included in each microplate. Negative control values were subtracted from enzyme assay values to estimate the conversion (0–100% compared with 1,2-cyclohexanediol). The experiments were conducted in triplicate.

In order to determine the enantiopreference of B1EPH2, pure (*R*)- and (*S*)-enantiomers of 2-phenyloxirane [(*R*)-styrene oxide, (*R*)-(**11**), and (*S*)-(+)-styrene oxide, (*S*)-(**11**)], (*R*)- and (*S*)-2-(chloromethyl)oxirane [(*R*)-(−)-epichlorohydrin, (*R*)-(**14**), and (*S*)-(+)-epichlorohydrin, (*S*)-(**14**)] and (*R*)- and (*S*)-1,2-ethyloxirane [(*R*)-(+)-1,2-epoxybutane, (*R*)-(**15**), and (*S*)-(−)-1,2-epoxybutane, (*S*)-(**15**)] were assayed using the adrenaline test. The reaction was carried out with 90 µg ml^−1^ B1EPH2 for both enantiomers of styrene oxide and with 1250 µg ml^−1^ B1EPH2 for both enantiomers of epichlorohydrin and 1,2-epoxybutane in sodium phosphate buffer pH 6.0 at 25°C for 10 min. The final concentration of substrate in the reactions was 50 m*M* for both enantiomers of styrene oxide and 100 m*M* for both enantiomers of epichlorohydrin and 1,2-epoxybutane. All substrates were diluted in DMSO. The estimated *E* value using pure enantiomers was measured in individual wells of the same microtiter plate, setting the endpoint as 10 min of reaction. The adrenochrome absorption was correlated indirectly to 1,2-diol formation using a calibration curve (sodium periodate consumption versus adrenochrome formation). Calculation of *E* from the initial enantiomeric reaction rates of both enantiomerically pure epoxides was based on the apparent rate *V*, where *E* is the enantiomeric ratio *V*_fast enantiomer_/*V*_slower enantiomer_ (Badalassi *et al.*, 2000 ▸).

### 2.3. Crystallization, X-ray data collection, structure determination, refinement and analysis   

B1EPH2 at a concentration of 10 mg ml^−1^ was initially subjected to approximately 500 different crystallization conditions. The drops were prepared using a HoneyBee 963 (Digilab Global) crystallization robot using 0.3 µl protein solution and 0.3 µl precipitant solution in 96-well sitting-drop plates with 40 µl well solution (LNBio-CNPEM, Campinas, Brazil). The plates were maintained in a Rock Imager 1000 (Formulatrix) equipped with UV–Vis light at 18°C for five months. After obtaining the initial crystal hits, the best conditions were manually optimized using the hanging-drop vapor-diffusion method in Linbro plates, and diffracting crystals were obtained in a condition consisting of 0.1 *M* sodium cacodylate pH 6.5, 1 *M* trisodium citrate using protein at a concentration of 12.5 mg ml^−1^ after 2–3 days. Further data on the crystallization methods and cryoprotection protocol are given in Table 2 ▸. Data were collected from B1EPH2 crystals on MX2 at Laboratório Nacional de Luz Síncrotron (LNLS), Campinas, Brazil. The X-ray diffraction data were processed using *XDS* (Kabsch, 2010 ▸) and scaled using *AIMLESS* (Evans & Murshudov, 2013 ▸) from the *CCP*4 suite (Winn *et al.*, 2011 ▸). The number of molecules in the asymmetric unit was estimated by *MATTHEWS_COEF* also from the *CCP*4 suite. The B1EPH2 structure was solved by molecular replacement using *Phaser* (McCoy *et al.*, 2007 ▸) from the *CCP*4 suite and the structure of epoxide hydrolase B from *Mycobacterium tuberculosis* (PDB entry 2zjf; James *et al.*, 2008 ▸) as a search model. The refinement of the B1EPH2 structure was carried out using *phenix.refine* (Afonine *et al.*, 2012 ▸) from the *Phenix* suite (Liebschner *et al.*, 2019 ▸). For enhancement, we used NCS restraints and one TLS group. Refinement in real space and visualization were performed using *Coot* (Emsley *et al.*, 2010 ▸). The stereochemical quality was ascertained by *MolProbity* (Chen *et al.*, 2010 ▸) and figures were prepared using *PyMOL* version 1.8 (Schrödinger).

## 3. Results and discussion   

### 3.1. Activity and enantiopreference of B1EPH2   

**Figure 1:**
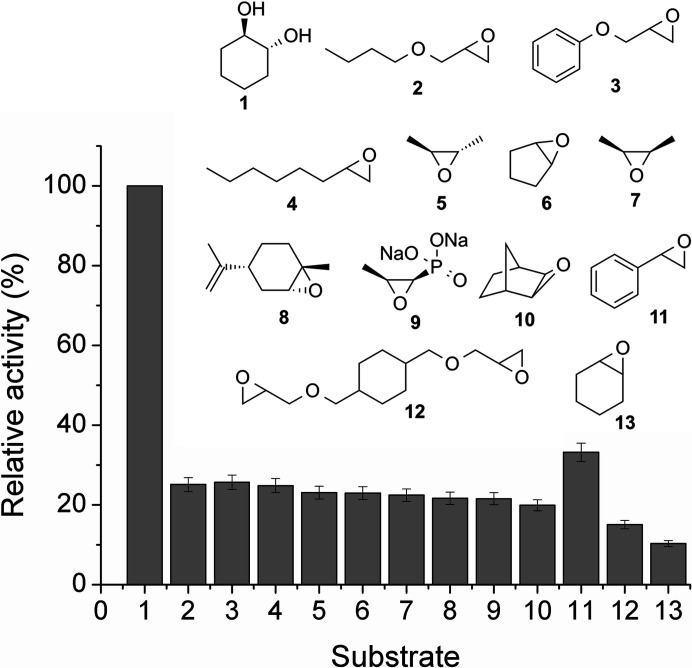
Relative activity of B1EPH2 against a broad range of substrates. The enzyme activity was measured using the adrenaline test protocol as described by Fluxá *et al.* (2008 ▸).

The adrenaline assay has been widely used to study the activities of EHs (Fluxá *et al.*, 2008 ▸). In this colorimetric test, compounds that are sensitive to sodium periodate such as the 1,2-diols formed by the enzymatic hydrolysis of epoxides, are chemically oxidized. The remaining periodate is retro-titrated with l-epinephrine, turning the colorless solution red by the formation of adrenochrome, which can be monitored at 490 nm in a miniaturized assay. To test the activity of B1EPH2 as epoxide hydrolase, 12 racemic epoxides with a diverse pattern of substituents were selected (Fig. 1 ▸). Remarkably, the enzyme showed activity from 20 µg ml^−1^ and was able to hydrolyse all assayed substrates. Additionally, it was recognized that the enzymatic activity increases according to the enzyme concentration, as expected. The relative conversion of epoxides to the corresponding 1,2-diols was compared with that of the positive control 1,2-cyclohexanediol (100% conversion). Under the assay conditions, the highest activities were observed for styrene oxide (**11**; 33% conversion), 1,2-epoxyoctane (**4**; 24% conversion) and phenyl glycidyl ether (**3**; 25% conversion). In general, the remaining substrates tested were hydrolysed to similar extents by the enzyme. The lowest activities measured were for 1,4-cyclohexene diglycidyl oxide (**12**) and cyclohexene oxide (**13**) (with 15% and 10% conversion, respectively; Fig. 1 ▸).

**Figure 2:**
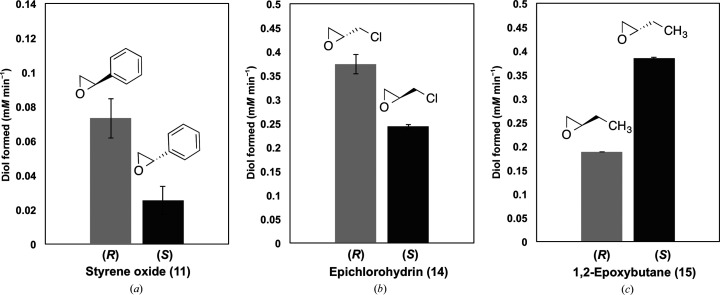
Preferential hydrolysis of enantiomeric epoxides by B1EPH2. The assay was performed with the (*R*)- and (*S*)-enantiomers of styrene oxide, epichlorohydrin and 1,2-epoxybutane using B1EPH2 as a biocatalyst. (*a*) 90 µg ml^−1^ B1EPH2 and 50 m*M* of both enantiomers of styrene oxide. (*b*) 1250 µg ml^−1^ B1EPH2 and 100 m*M* of both enantiomers of epichlorohydrin. (*c*) 1250 µg ml^−1^ B1EPH2 and 100 m*M* of both enantiomers of 1,2-epoxybutane.

In order to identify the enantiopreference of B1EPH2, the (*R*)- and (*S*)-enantiomers of 2-phenyloxirane (styrene oxide; **11**), 2-(chloromethyl)oxirane (epichlorohydrin; **14**) and 1,2-ethyloxirane (epoxybutane; **15**) were also evaluated using the adrenaline assay. This experiment showed that the enzyme is slightly more selective for the hydrolysis of (*R*)-(+)-styrene oxide (estimated *E* = 2.9; Table 3 ▸) than the (*S*)-enantiomer (Fig. 2 ▸ *a*). B1EPH2 is also enantioselective for epichlorohydrin and 1,2-epoxybutane, and preferentially hydrolyses (*R*)-(−)-epichlorohydrin (Fig. 2 ▸ *b*; estimated *E* = 1.5; Table 3 ▸) and (*S*)-(−)-1,2-epoxybutane (Fig. 2 ▸ *c*; estimated *E* = 2.0; Table 3 ▸). These results are similar to those observed for the EH from *Phanerochaete chrysosporium* (Li *et al.*, 2009 ▸), which in contrast has a greater preference for the hydrolysis of (*R*)-(+)-styrene oxide but a very limited selectivity for the (*R*)- or (*S*)-enantiomers of epichlorohydrin or 1,2-epoxybutane (*E* values were not estimated). These assays indicate that B1EPH2 can hydrolyse both (*R*)- and (*S*)-enantiomers to different extents, with a difference of between 1.5-fold and threefold for specific enantiomers according to Fig. 2 ▸. However, the estimated enantiomeric ratio (*E*) observed for all compounds is modest. The main difference between experiments using separate enantiomerically pure probes (estimated *E* values) and the true *E* values is the absence of enantiomeric competition for the same enzymatically active site, which can lead to estimated *E* values that can be far from the true values (Chen *et al.*, 1982 ▸; Janes & Kazlauskas, 1997 ▸; Mantovani *et al.*, 2008 ▸). The estimated *E* values were calculated based on the apparent rate (*V*) of (*R*)- and (*S*)-1,2-diol formation after 10 min of reaction (Table 3 ▸). Compared with the enzyme preferences given above, it is possible to suggest that the nucleophilic attack by water occurs in the less hindered position, as in other retaining enzymes, and therefore the stereochemistry of the substrates drives the enantioselectivity. For styrene oxide, the preference for the opposite chiral isomer could be owing to better positioning of the aromatic substituent inside the active-site pocket. However, further investigation, including site-directed mutagenesis, is necessary to determine the basis for the stereoselectivity of this enzyme.

### 3.2. Structure of B1EPH2   

**Figure 3:**
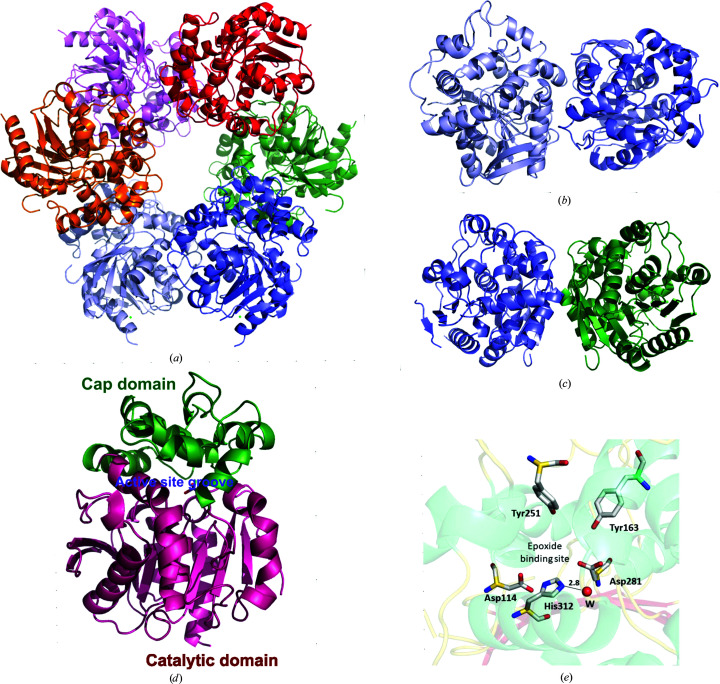
Overall structure of B1EPH2. (*a*) Quaternary structure of B1EPH2. (*b*) Contacts between protomers *A* and *B*. (*c*) Contacts between protomers *B* and *C*. (*d*) Monomer of B1EPH2 indicating the cap domain (green) and catalytic domain (purple) and the active-site groove between them (blue). (*e*) The active site of B1EPH2, indicating the essential residues for catalysis. W is a water molecule.

The B1EPH2 crystals belong to space group *P*4_1_22 and diffract to a maximum resolution of 2.2 Å. Further data on X-ray data processing are summarized in Table 4 ▸. The structure of B1EPH2 was solved by molecular replacement using the structure of the soluble epoxide hydrolase B from *M. tuberculosis* (MtEHB; James *et al.*, 2008 ▸), which shares an identity of about 49%. The final model of B1EPH2 consists of 8049 non-H atoms and has an *R* factor and *R*_free_ of 18.2% and 24.0%, respectively (Table 5 ▸). The atomic coordinates and structure factors have been deposited in the PDB as entry 6unw. The asymmetric unit of B1EPH2 contains three protomers, each with a molecular mass of 37 kDa, which generate a hexamer through twofold crystallographic symmetry; consequently, the protomers in the asymmetric unit are connected by a threefold axis (Fig. 3 ▸ *a*), supporting the results obtained from analytical gel filtration, in which the protein eluted with a retention time corresponding to 252 kDa, matching a hexameric quaternary structure (Supplementary Fig. S1).

The r.m.s.d. for superposition of the three different protomers in the asymmetric unit is about 0.3 Å, indicating that they are very similar and there are no large conformation changes between them. This is, at least to our knowledge, the first description of a hexameric soluble epoxide hydrolase, in contrast to several dimeric soluble EHs (Mowbray *et al.*, 2006 ▸; Gomez *et al.*, 2004 ▸; James *et al.*, 2008 ▸; Argiriadi *et al.*, 1999 ▸) and a monomeric soluble EH characterized by our research group (Wilson *et al.*, 2017 ▸; de Oliveira, Adriani *et al.*, 2019 ▸). The hexamer is stabilized predominantly by hydrophobic inter­actions, with similar interaction surfaces of 724 and 708 Å^2^ between protomers *A* and *B* and protomers *B* and *C*, respectively, and with a further nine and 14 ionic interactions observed between protomers *A* and *B* and protomers *B* and *C*, respectively (Supplementary Fig. S2). Interestingly, the interaction surface between protomers *A* and *B* is similar to that observed in the dimeric structure of the soluble EH from *M. tuberculosis* (Fig. 3 ▸ *b*), while the interaction surface between protomers *B* and *C* (Fig. 3 ▸ *c*) has not been observed in any other dimeric soluble EHs, including human epoxide hydrolase (hEPH; James *et al.*, 2008 ▸).

Each protomer of B1EPH2 adopts a classical α/β-hydrolase fold, which consists of a cap domain, which is the more flexible and less conserved region, constituted of six α-helices, and a catalytic domain. The catalytic domain is highly conserved among different soluble EHs and adopts a typical eight-stranded β-sheet with two antiparallel and six parallel strands, which are sandwiched by four α-helices, two on one side and two on the other side of the β-sheet (Fig. 3 ▸ *d*).

Similarly to other soluble EHs, the active site of B1EPH2 is located in a large groove of about 35 Å between the cap and hydrolase domains, and residues from both domains participate in catalysis. The conserved catalytic triad is placed in the hydrolase domain and is constituted by Asp114, His312 and Asp281. On the other hand, two conserved tyrosine residues, Tyr163 and Tyr251, are located in the cap domain (Fig. 3 ▸ *e*). The role of these active-site residues has already been well characterized in various epoxide hydrolases and the mechanism of catalysis of B1EPH2 should be similar to those described previously (Nardini *et al.*, 2001 ▸).

#### 3.2.1. Electron density in the active site of B1EPH2   

**Figure 4:**
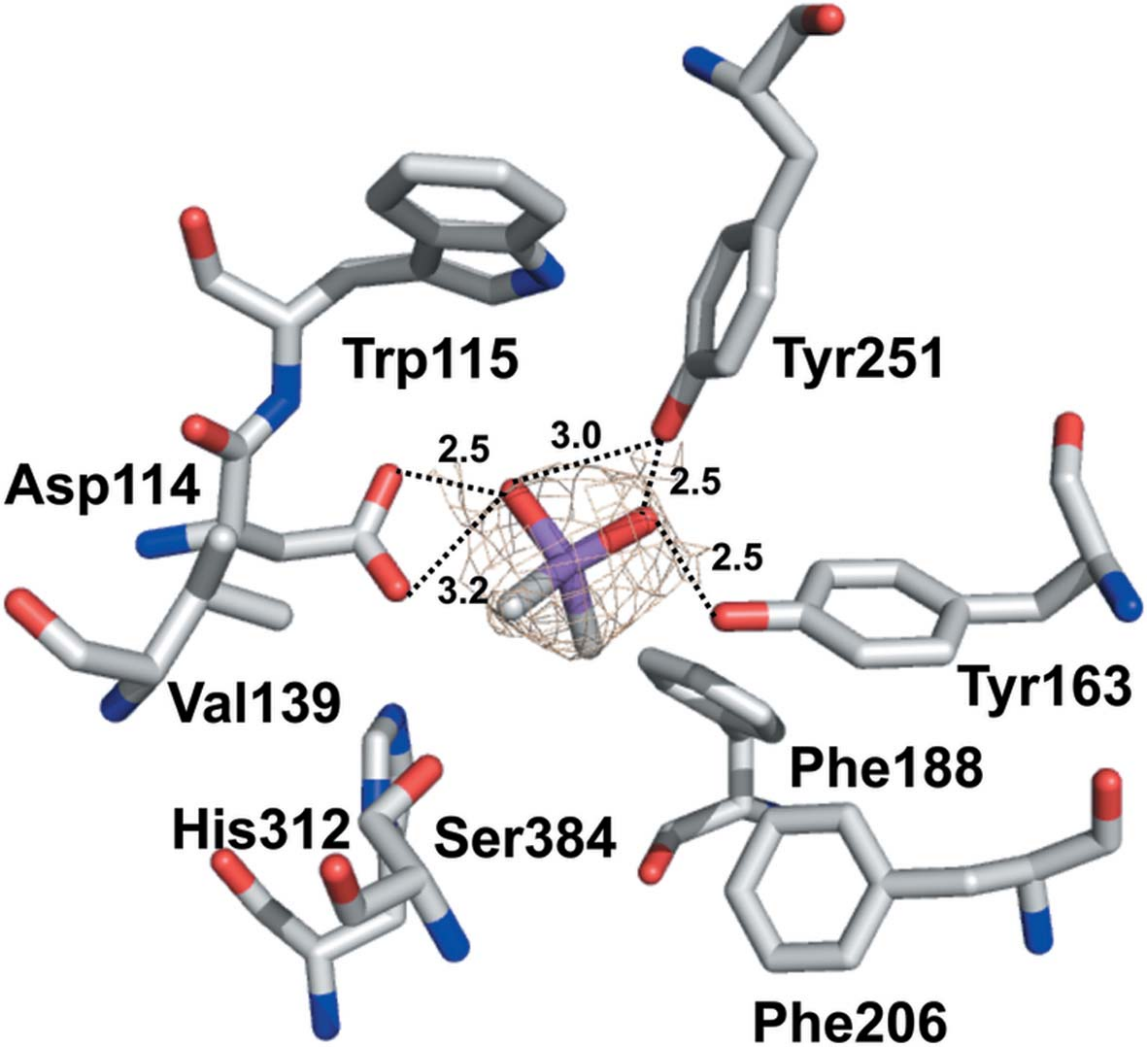
Electron density observed in the active site of B1EPH2 possibly occupied by a cacodylate molecule. The dotted lines are hydrogen-bond interactions between the cacodylate molecule and active-site residues. Distances are given in Å.

Although several attempts were made to obtain the structure of B1EPH2 in the presence of valpromide, a known epoxide hydrolase inhibitor, our results do not indicate any electron density corresponding to this ligand. There is, however, electron density in the active site of B1EPH2 which has been modeled as cacodylate. Although cacodylate has not been reported to be an inhibitor of epoxide hydrolases, it could bind to the active site of the enzyme owing to the high concentration of this molecule used in the crystallization condition. Fig. 4 ▸ shows cacodylate modeled in protomer *A* of B1EHP2. The cacodylate makes interactions with key residues of the active site, including Asp114 from the catalytic domain and Tyr116 and Tyr251 from the cap domain. Further studies are necessary to determine whether cacodylate could be an authentic epoxide hydrolase inhibitor.

#### 3.2.2. B1EPH2 and other microbial EHs   

**Figure 5:**
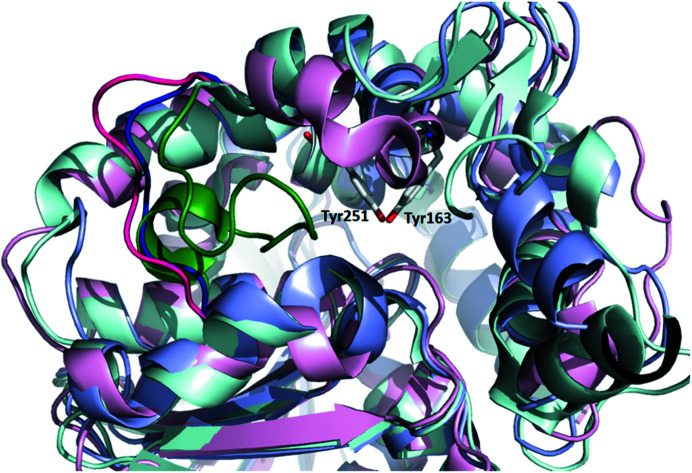
B1EPH2 active site showing a superposition of the secondary elements of B1EPH2 (blue), MtEHB (green) and hEPH (pink). The loop corresponding to residues 142–148 of B1EPH2 is shown in darker colors.

As observed for EHB from *M. tuberculosis*, B1EPH2 has several hydrophobic aromatic residues in the catalytic groove that could drive the specificity of this enzyme. Although several of these residues are conserved when B1EPH2 is compared with MtEHB, other residues such as Tyr188, Leu193, Phe206, Val164, Phe162 and Phe165 are not conserved and should alter the volume and charge of the active site. Besides, the active site of B1EPH2 is larger than that of MtEHB because of an insertion between Gly133 and Pro143 in MtEHB that protrudes into the active-site cavity and considerably reduces its volume (129 Å^3^). In contrast, similar to that of human hEPH, the B1EPH2 active site has a volume of approximately 630 Å^3^ according to the *POCASA* webserver (Yu *et al.*, 2010 ▸). Interestingly, although B1EPH2 has a higher similarity to MtEHB (49%), as they are from phylogenetically related microorganisms, the active-site cavity volume of B1EPH2 is much more similar to that of the human epoxide hydrolase (36% similarity), which explains its high acceptance of larger substrates (Fig. 5 ▸). However, we could not observe any correlation between the structure of B1EPH2 and its high activity with several specific alternative substrates assayed here.

## 4. Conclusions   

This work shows the structural and catalytic features of a putative α/β-epoxide hydrolase encoded by the genome of *Streptomyces* sp. CBMAI 2042. A genome-guided strategy facilitated the annotation of the gene as *b1eph2.* Gene cloning and expression led to the production of B1EPH2, which is the first hexameric EH to be characterized. Enzymatic assays confirmed the activity of B1EPH2 as an epoxide hydrolase that accepts a wide range of substrates. The enzyme is slightly enantioselective towards several substrates. Based on structural studies, we speculate that B1EPH2 should have a similar specificity for longer-chain substituted substrates to that observed for human epoxide hydrolase, despite its highest sequence similarity being to *M. tuberculosis* EHB, which has an insertion that protrudes into the active site and decreases the size of its cavity. Based on this, the active site of B1EPH2 is comparable to that of the human enzyme in volume. However, B1EPH2 also has several nonconserved amino-acid residues in the active site that could drive its specificity to particular substrates. Further experiments such as site-directed mutagenesis of hydrophobic residues in the B1EPH2 active site will be conducted to tackle the specificity and tune the enantio­selectivity of this enzyme and its potential as a biocatalyst for chemoenzymatic applications.

## 5. Related literature   

The following references are cited in the supporting information for this article: Laskowski *et al.* (2018 ▸).

## Supplementary Material

PDB reference: B1EPH2, 6unw

Supplementary Figures. DOI: 10.1107/S2059798320010402/ud5015sup1.pdf

## Figures and Tables

**Table 1:** Macromolecule-production information

Source organism	*Streptomyces* sp. CBMAI 2042
Forward primer	GACCATATGACGACGACCCCACCACC
Reverse primer	CTCGAATTCCGCAGCCCGTCCAGCCAC
Expression vector	pET-29b(+)
Expression host	*E. coli* BL21(DE3)
Complete amino-acid sequence of the construct produced	VPQPPTDDPTTPAEKGAVHRLVDTPGGRIHLVEQGTGPLVLLVHGFPESWYSWRHQLPALAAAGYRAAAIDVRGYGRSAKPAATDAYRMLAHVADNTAVVHALGEETATVVGHDWGSPIAANSALLRPDVFTAVGLLSVPYAPRGEHRPTDGFARIGGDEEFYVSYFQAPGRAEAEIERDVRGWLAGFYTGLTGGALTPEEHGRLFFVPPGAHLADRFPTGPLPAWLTEADLDVYSGEFERSGLTGALNRYRNVDRDWEDLAAWHGAPITQPSLFIGGALDASTTWMADALDAYPATLPGLSAAHILEGCGHWIQQERPDEVNRLLTQWLDGLR

**Table 2:** Crystallization conditions for B1EPH2 and cryoprotection protocol

Method	Hanging-drop vapor diffusion
Plate type	Linbro plates
Temperature (K)	293
Protein concentration (mg ml^−1^)	12.5
Buffer composition of protein solution	50 m*M* HEPES pH 8.0, 300 m*M* NaCl
Composition of reservoir solution	0.1 *M* sodium cacodylate pH 6.5, 1 *M* trisodium citrate
Volume and ratio of drop	1:1
Volume of reservoir (ml)	400
Cryoprotectant	30% ethylene glycol, 70% reservoir solution

**Table 3:** Apparent reaction rates (*V*) of 1,2-diol formation during the hydrolysis of epoxides **11**, **14** and **15** promoted by B1EPH2

	*V*_(*R*)_ (mmol min^−1^)	*V*_(*S*)_ (mmol min^−1^)	Estimated *E* †
1-Phenylethane-1,2-diol	0.073 (±0.011)	0.025 (±0.008)	2.9
3-Chloropropane-1,2-diol	0.373 (±0.020)	0.243 (±0.003)	1.5
Butane-1,2-diol	0.188 (±0.0006)	0.385 (±0.002)	2.0

† *E* is the enantiomeric ratio between *V*_(*R*)_ and *V*_(*S*)_.

**Table 4:** Data-collection and processing statistics for B1EPH2 Values in parentheses are for the outer shell.

Diffraction source	MX2, LNLS
Wavelength (Å)	1.48
Temperature (K)	100
Detector	PILATUS 2M
Rotation range per image (°)	0.2
Total rotation range (°)	360
Exposure time per image (s)	2
Space group	*P*4_1_22
*a*, *b*, *c* (Å)	106.7, 106.7, 233.0
Resolution range (Å)	48.50–2.21 (2.26–2.21)
Total No. of reflections	1184544 (50856)
No. of unique reflections	67798 (42806)
Completeness (%)	99.5 (95.0)
Multiplicity	17.5 (11.9)
〈*I*/σ(*I*)〉	22.0 (2.1)
CC_1/2_	0.999 (0.706)
*R*_p.i.m._	0.041 (0.506)
*R*_r.i.m._ †	0.124 (12.43)
Overall *B* factor from Wilson plot (Å^2^)	29.1

† Estimated *R*_r.i.m._ = *R*_merge_[*N*/(*N* − 1)]^1/2^, where *N* is the data multiplicity.

**Table 5:** Structure-solution and refinement statistics for B1EPH2 Values in parentheses are for the outer shell.

Resolution range (Å)	48.52–2.21 (2.29–2.21)
Completeness (%)	94.9 (96.7)
No. of reflections, working set	64933 (6476)
No. of reflections, test set	3267 (362)
Final *R*_cryst_	0.187 (0.276)
Final *R*_free_	0.227 (0.326)
No. of non-H atoms	
Total	8098
Protein	7485
Ligand (ions and cacodylate)	20
Solvent	593
R.m.s. deviations	
Bond lengths (Å)	0.009
Angles (°)	1.25
Average *B* factors (Å^2^)	
Protein	39.60
Solvent	41.57
Ligands	61.43
Ramachandran plot	
Most favored (%)	97.20
Allowed	2.07
Outliers (%)	0.73
Rotamer outliers (%)	0.68
Clashscore	7.39
No. of TLS groups	1
