# Supplementary material for: An epoxide hydrolase from endophytic Streptomyces shows unique structural features and wide biocatalytic activity
Source: Acta Crystallogr D Struct Biol. 2020 Aug 17;76(Pt 9):868–75. doi: 10.1107/S2059798320010402 (PMC7466753; doi:10.1107/S2059798320010402)
Supplement: Supplementary file 1 [file d-76-00868-sup1.pdf]

STRUCTURAL BIOLOGY

Volume 76 (2020)

Supporting information for article:

An epoxide hydrolase from endophytic *Streptomyces* shows unique structural features and wide biocatalytic activity

Gabriela D. Tormet-González, Carolina Wilson, Gabriel Stephani de Oliveira, Jademilson Celestino dos Santos, Luciana G. de Oliveira and Marcio Vinicius Bertacine Dias

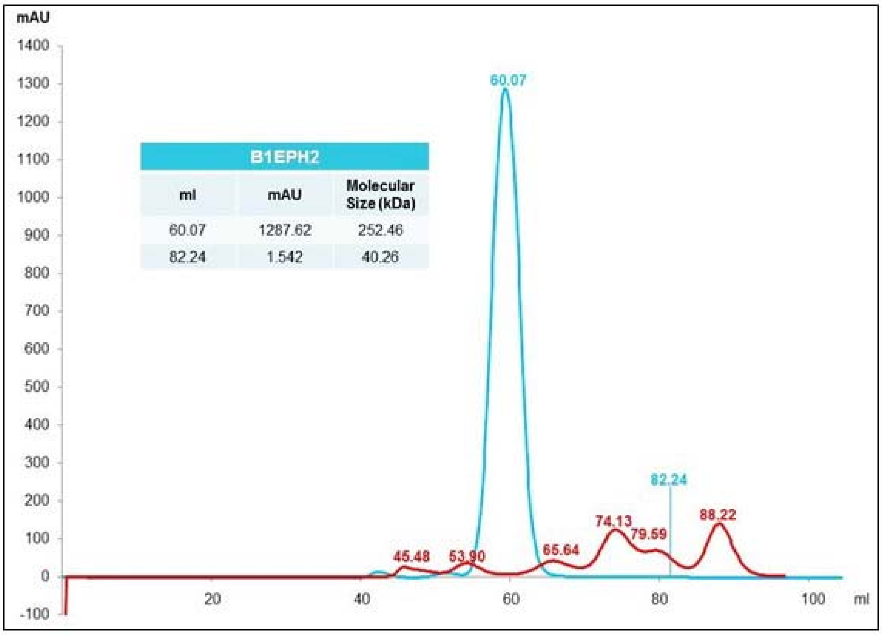

Figure S1 Elution profile for B1EPH2. The red line shows the peaks of proteins with known molecular weight used to calibrate a Superdex 200 (16/60 column). The proteins used includes carbon anhydrase, ovalbumin, conalbumin, aldolase and ferritin. The blue line shows the retention time for B1EPH2. To obtain the molecular weight of B1EPH2 we have used a linear regression by Origin. The left inset shows the estimated molecular size (kDa) for the B1EPH2. (inset) A majority peak is observed at 60.07 mL and correspond a similar mass of a hexamer of B1EPH1 and a negligible peak could be observed at 82.24 mL.

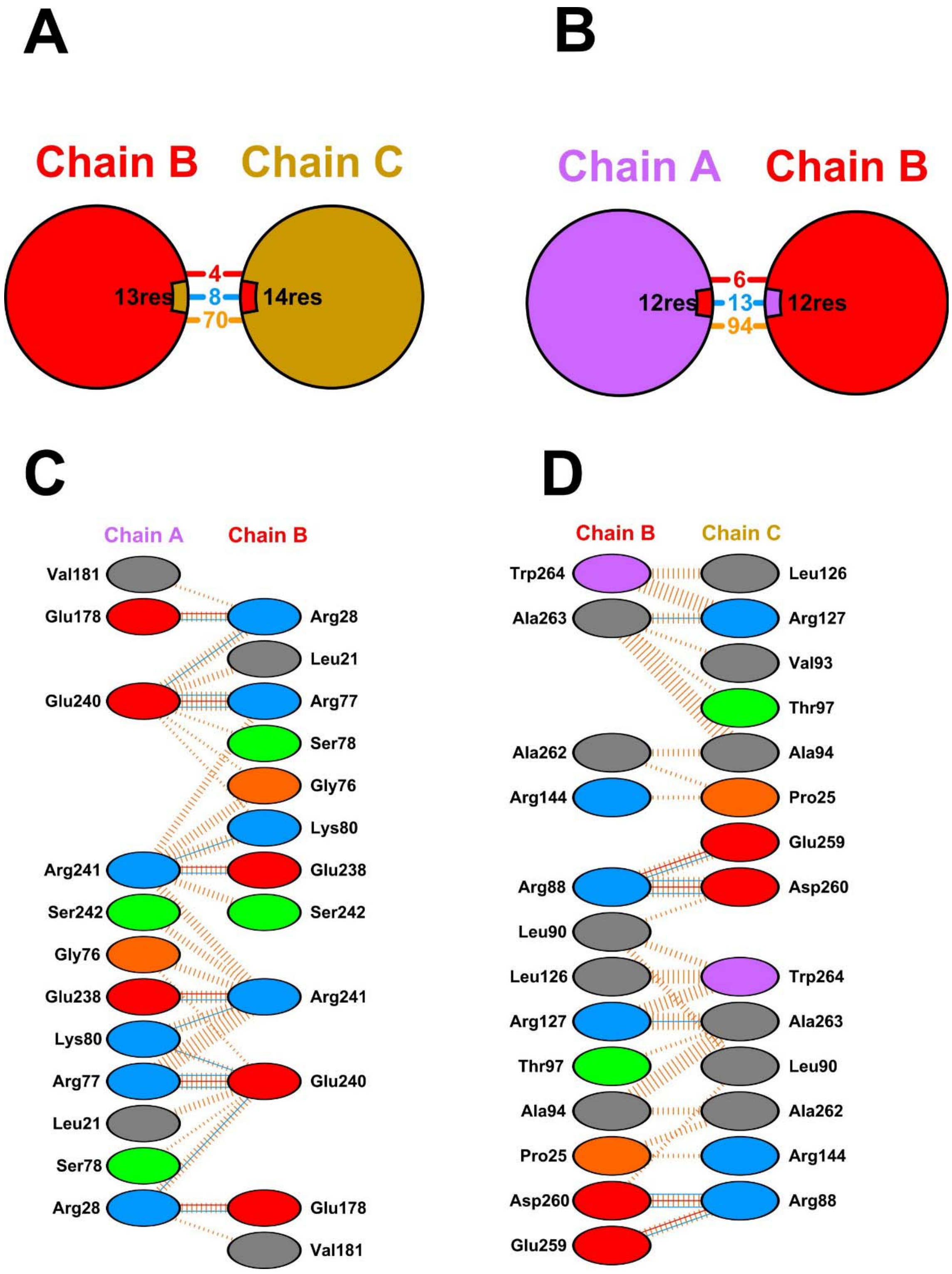

Analysis of interface of different protomers of B1EPH2. A) number of interactions between protomers A and B. (B) number of interactions between protomers B and C. Red line is the ionic interaction, blue is the hydrogen interaction and yellow is the nonbonding interactions. The area in the circle corresponding the surface of interaction C) residues involved in the interaction of protomers A and B and D) residues involved in the interaction of monomers B and C. The analysis was performed using the online server PDBsum (Saskowski et al., 2018).

Reference

Laskowski, R. A., Jabłońska, J., Pravda, L., Vařeková, R. S. & Thornton, J. M. (2018). *Protein Science* **27**, 129-134.
